# Identification of *in vivo* Essential Genes of *Vibrio vulnificus* for Establishment of Wound Infection by Signature-Tagged Mutagenesis

**DOI:** 10.3389/fmicb.2019.00123

**Published:** 2019-02-01

**Authors:** Kohei Yamazaki, Takashige Kashimoto, Mio Morita, Takehiro Kado, Kaho Matsuda, Moeko Yamasaki, Shunji Ueno

**Affiliations:** Laboratory of Veterinary Public Health, School of Veterinary Medicine, Kitasato University, Towada, Japan

**Keywords:** comprehensive analysis, signature-tagged mutagenesis, *Vibrio vulnificus*, virulence genes, wound infection

## Abstract

*Vibrio vulnificus* can cause severe necrotic lesions within a short time. Recently, it has been reported that the numbers of wound infection cases in healthy hosts are increasing, for which surgical procedures are essential in many instances to eliminate the pathogen owing to its rapid proliferation. However, the mechanisms by which *V. vulnificus* can achieve wound infection in healthy hosts have not been elucidated. Here, we advance a systematic understanding of *V. vulnificus* wound infection through genome-wide identification of the relevant genes. Signature-tagged mutagenesis (STM) has been developed to identify functions required for the establishment of infection including colonization, rapid proliferation, and pathogenicity. Previously, STM had been regarded to be unsuitable for negative selection to detect the virulence genes of *V. vulnificus* owing to the low colonization and proliferation ability of this pathogen in the intestinal tract and systemic circulation. Alternatively, we successfully identified the virulence genes by applying STM to a murine model of wound infection. We examined a total of 5418 independent transposon insertion mutants by signature-tagged transposon mutagenesis and detected 71 clones as attenuated mutants consequent to disruption of genes by the insertion of a transposon. This is the first report demonstrating that the pathogenicity of *V. vulnificus* during wound infection is highly dependent on its characteristics: flagellar-based motility, siderophore-mediated iron acquisition system, capsular polysaccharide, lipopolysaccharide, and rapid chromosome partitioning. In particular, these functions during the wound infection process and are indispensable for proliferation in healthy hosts. Our results may thus allow the potential development of new strategies and reagents to control the proliferation of *V. vulnificus* and prevent human infections.

## Introduction

*Vibrio vulnificus* is a Gram-negative halophilic bacterium and opportunistic human pathogen that causes primary septicemia and wound infections (Oliver, 2005, 2015; Menon et al., 2014). Epidemiological studies show that the incidence of *V. vulnificus* wound infection, which can result from exposure to seawater or through handling marine products, is increasing (Oliver, 2005). This is primarily due to rising seawater temperatures caused by global warming, which are essential for the rapid proliferation of this pathogen (Vezzulli et al., 2013; Oliver, 2015). The wound infection can result in lethal septicemia or require surgery to prevent extensive tissue damage and remove bacteria from the infection sites. However, the mechanisms of pathogenesis in *V. vulnificus* wound infection remain to be elucidated.

To date, *in vivo* expression technology (IVET), *in vivo*-induced antigen technology (IVIAT), and signature-tagged mutagenesis (STM) have been used to identify virulence factors of *V. vulnificus* that are functionally expressed *in vivo*, focusing on primary septicemia (Kim et al., 2003; Lee et al., 2007; Yamamoto et al., 2015). Both IVET and IVIAT comprise positive selection methods, which can identify genes whose expression is enhanced *in vivo* after excluding constitutively expressed genes (Mahan et al., 1993; Kim et al., 2003; Lee et al., 2007). In contrast, STM is a negative selection method that can detect essential genes for *in vivo* proliferation and functionally deficient mutants (Hensel et al., 1995; Yamamoto et al., 2015). However, although harvesting sufficient bacterial number from hosts as an output pool is indispensable for the negative selection of mutants, *V. vulnificus* is not able to efficiently colonize and proliferate in mouse models of primary septicemia (Lin et al., 2014; Kashimoto et al., 2015). Thus, STM had been considered not to be suitable for the identification of virulence genes of *V. vulnificus*. However, we could show that a sufficient number of bacteria required for the STM can be collected from a murine model of wound infection and that the lethal outcomes cannot be prevented if allowing sufficient proliferation by *V. vulnificus* in muscle tissue (Yamazaki et al., 2017). In addition, recent epidemiological report and our previous report suggested that STM is an adequate method to identify essential genes for *V. vulnificus* proliferation only in a murine model of wound infection. Oliver (2015) reported that 94% of patients with primary septicemia have one or more underlying disease(s), whereas over 80% of individuals presenting with *V. vulnificus* wound infection have no underlying diseases. This suggests that soft-tissues in wound infection constitute an environment for bacterial proliferation that is significantly different from the intestinal tract or systemic circulation affected in primary septicemia.

Virulence genes are preferentially expressed during infection. Both in primary septicemia and wound infection of *V. vulnificus*, the multifunctional-autoprocessing repeats-in-toxin (MARTX) toxin is known to be indispensable for pathogenicity and colonization (Chung et al., 2010; Lo et al., 2011; Jeong and Satchell, 2012). Toxic effects of the MARTX toxin are exerted in a cell contact-dependent manner and can kill host cells, leading to bacterial resistance against neutrophils (Kim et al., 2008). Moreover, the incubation period for *V. vulnificus* wound infection cases averages only 16 h, which is much shorter than that of primary septicemia (Oliver, 2015). These findings encourage the hypothesis that *V. vulnificus* carries mechanisms for immune evasion, colonization, and rapid proliferation especially in wound infection. These processes would be mediated by factors that are constitutively expressed and preemptively function in early stages of infection, underlying its pathogenic ability to establish wound infection within a short time. To test this hypothesis, we applied STM to a murine model of wound infection, which was expected to resolve the problems of low-efficiencies in harvesting the pathogen from systemically infected animals, and identified the genes and functions required by *V. vulnificus* for efficient proliferation in wound infection.

## Materials and Methods

### Animals

We utilized five-week-old female C57BL/6 mice (Charles River Laboratories Japan, Yokohama, Japan) for the animal experiments. The mice were housed in a controlled environment with a 12:12-h light-dark cycle and were fed rat chow MF (Oriental Yeast, Tokyo, Japan) and tap water. Ambient temperature during the study was maintained at about 23°C.

### Ethics Statement

All animal studies were carried out in strict accordance with the Guidelines for Animal Experimentation of the Japanese Association for Laboratory Animal Science. The animal experimentation protocol was approved by the President of Kitasato University based on the judgment of the Institutional Animal Care and Use Committee of Kitasato University (Approval No. 15–156).

### Bacterial Strains

*Vibrio vulnificus* CMCP6 is a clinical isolate from a male septicemic patient at the Chonnam National University Hospital, South Korea (Kim et al., 2003; Tan et al., 2014), and kindly provided by Dr. Joon Haeng Rhee (Chonnam National University, South Korea). The complete genome sequence of CMCP6 is available in some databases, Joint Genome Institute^1^ and Kyoto Encyclopedia of Genes and Genomes (KEGG)^2^. *Escherichia coli* BW19795 with signature-tagged mini-Tn5Km2 in pUT were used for conjugation of transposons to *V. vulnificus*. Δ*pomA* is a non-motile strain used as a control strain for the swarming assay (Gulig et al., 2009). *V. vulnificus* E4 is unencapsulated strain (translucent colony morphology) and was used as a control strain for colony morphology. This strain was isolated from seafood in Florida, and kindly provided by Dr. Shinichi Miyoshi (Okayama University, Japan) (Kashimoto et al., 2003).

### STM

**Figure 1:**
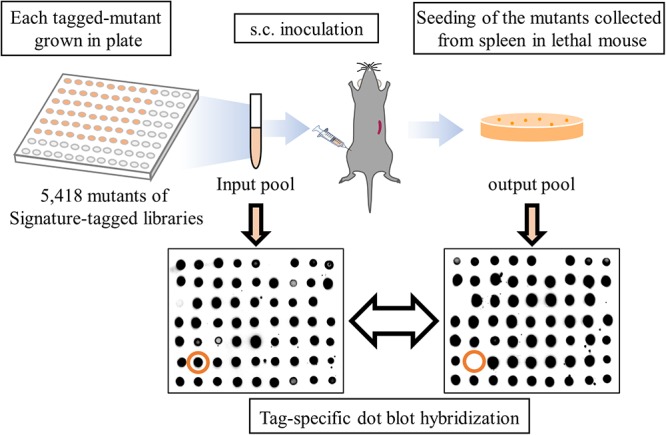
Scheme of STM application to a murine model of *V. vulnificus* wound infection. A result of tag-specific dot blot hybridization for the input pool and output pool following a round of STM. Orange circles indicate tags detected in the input pool but not in the output pool.

The scheme of STM is shown in Figure 1. The construction of a library containing 63 mutants with a transposon tagged by a unique sequence was performed as previously described (Yamamoto et al., 2015). Briefly, *E. coli* BW19795 with signature-tagged mini-Tn5Km2 in pUT were combined with *V. vulnificus* on a nitrocellulose Hybond C membrane (GE Healthcare) for conjugation, placed onto an M9 agar plate, and incubated at 25°C (Forsyth and Kushner, 1970; de Lorenzo et al., 1990; Pobigaylo et al., 2006). The bacterial suspension was plated onto Thiosulfate-Citrate-Bile Salts-Sucrose (TCBS) agar containing 100 μg/ml kanamycin and incubated overnight at 37°C for selection (Pfeffer and Oliver, 2003). Each signature-tagged transposon insertion mutants of *V. vulnificus* were grown in Luria-Bertani (LB) medium containing 100 mg/ml of kanamycin for 12 h at 37°C in 96 well plate separately. We checked each bacterial growth by measuring optical density at 600 nm (OD600) with a microplate reader (Sunrise/TECAN Japan, Kanagawa, Japan), and the mutants were pooled, washed with LB medium without any anti-biotics, and used as an input pool. Mice were subcutaneously inoculated with 10^6^ CFU of the input pool into right caudal thighs (Kashimoto et al., 2005). The infected mice were carefully monitored and sacrificed by sevoflurane (Wako pure chemical industries, Osaka, Japan) inhalation 12 to 24 h post-infection when they displayed critical symptoms that are directly associated with death, such as deep hypothermia, and the output pool was collected from murine spleens (Figure 1). Selection of attenuated mutants by STM was performed in triplicate for each of 86 libraries.

For tag-specific dot hybridization, 10 μM of the target DNA, comprising the signature-tagged sequence region of each transposon, was blotted onto Hybond-N+ membrane (GE Healthcare) and fixed with CL-1000 Ultraviolet Crosslinkers (UVP, Upland, CA, United States). DNA probes were amplified by polymerase chain reaction (PCR) with Dig-labeled primers: Dig 1 (5′-Dig-CAT GGT ACC CAT TCT AAC-3′) and Dig 2 (5′-Dig-TAC CTA CAA CCT CAA GCT-3′). PCR was performed in a 50-μl reaction mix containing 25 μl of 2 × PCR Buffer for KOD FX Neo, 5 μl of 2 mM dNTPs, 2 μl of primer mix (0.3 μM final concentration of each primer), 0.5 μl KOD FX Neo polymerase (0.5 unit, Toyobo, Osaka, Japan), 1 μl DNA template (about 200 ng genomic DNA), and distilled water. Thermal cycling conditions were as follows: (i) 5 min at 94°C; and (ii) 25 cycles of 15 s at 94°C, 45 s at 59°C, and 10 s at 68°C. The hybridization processes were performed in a hybridization oven (MHS-200e/eyelaco, Tokyo, Japan) (Yamamoto et al., 2015).

The transposon inserted sequence was determined via arbitrarily primed PCR using specific primers targeting the transposon: Rev consensus2 I-out 1st (5′-CCA TGG GTA AGA TTG GTT CGA A-3′), consensus1 I-out 1st (5′-GGT ACC TAC AAC CTC AAG CT-3′), and Rev consensus1 I-out for 2nd (5′-AGC TTG GTT AGA ATG GGT ACC-3′), and random primers targeting the *V. vulnificus* genome: Arb1 (5′-GGC CAC GCG TCG ACT AGT CAN NNN NNN NNN GAT AT-3′), Arb3 (5′-GGC CAC GCG TCG ACT AGT CAN NNN NNN NNN TTC AA-3′), Arb4 (5′-GGC CAC GCG TCG ACT AGT CAN NNN NNN NNN CCA CG-3′), Arb5 (5′-GGC CAC GCG TCG ACT AGT CAN NNN NNN NNN ACT GA-3′), Arb6 (5′-GGC CAC GCG TCG ACT AGT CAN NNN NNN NNN ACG CG-3′), Arb7 (5′-GGC CAC GCG TCG ACT AGT CAN NNN NNN NNN TGG CA-3′), and Arb for 2nd (5′-GGC CAC GCG TCG ACT AGT CA-3′). Thermal cycling conditions of first round were as follows: (i) 5 min at 95°C; (ii) 6 cycles of 20 s at 95°C, 20 s at 30°C, and 2 min at 72°C; (iii) 30 cycles of 20 s at 95°C, 20 s at 45°C, and 2 min at 72°C; and (iv) 5 min at 72°C. PCR was performed in a 50-μl reaction mix containing 5 μl of 10 × PCR Buffer for Paq5000, 2 μl of 2 mM dNTPs, 2 μl of primer mix (0.5 μM final concentration of each primer), 0.5 μl Paq5000 polymerase (0.5 unit, Stratagene, CA, United States), 1 μl DNA template (about 200 ng genomic DNA), and distilled water. Thermal cycling conditions of second round were as follows: (i) 30 s at 95°C; (ii) 30 cycles of 20 s at 95°C, 30 s at 55°C, and 4 min at 68°C; and (iii) 5 min at 68°C. PCR was performed in a 50-μl reaction mix containing 5 μl of 10 × PCR Buffer for Taq DNA polymerase with ThermoPol, 5 μl of 2 mM dNTPs, 2 μl of primer mix (0.5 μM final concentration of each primer), 0.5 μl Taq DNA polymerase with ThermoPol (0.5 unit, New England Biolabs, MA, United States), 1 μl the PCR product of the first round, and distilled water. The DNA sequences of the PCR products were determined by using the Fasmac sequencing service (Atsugi, Japan) and used to search for sequence homologies in the KEGG.

### Construction of a *pomA* Mutant and a *cheY* Mutant

To delete a 762-bp DNA fragment of *pomA*, approximately 1000 bp of each 5′ and 3′ of flanking regions of PomA encoding gene were amplified by PCR with the primers: *pomA* Up Fw (5′-GGG GTG ACG CCA AAG TAT ATG GTG AAT GCG AGC GTT TG-3′), *pomA* Up Rev (5′-TCT TAA GTT TGC TTC CCT CAT GCT ATT TTC CGA TTT ACC GC-3′), *pomA* Down Fw (5′-AAC GGG AGG AAA TAA TCA CGG GAG TAT GTG ATG GAT GAC G-3′), *pomA* Down Rev (5′-CTT AAC GGC TGA CAT GGG GAC GAG CAT TTC TGC TCA TC-3′), and *pomA* Flag Rev (5′-TTA TTT CCT CCC GTT TTA GAT TAC AAG GAT GAC GAC GAT AAG ATC GTT GAT ATC GAG GGC AC-3′). The amplified DNA were cloned into the suicide vector pYAK1, which contains the chloramphenicol resistant gene and *sacB* gene conferring sensitivity to sucrose (Kodama et al., 2002).

A 2859-bp DNA fragment including *cheY* was amplified using specific primers: *cheY* up Fw (5′-CTG CAG TGA ATG TGA GCC TCG AAC TC-3′) and *cheY* down Rev (5′-GGA TCC GCA TTG AGA AGA TCC CTG TC-3′). It cloned into the pGEM-T Easy vector (Promega, WI, United States). To delete a 381-bp DNA fragment of *cheY*, the plasmid was amplified by inverse PCR using specific primers: Inv Fw (5′-TTA GGG CCC CAA AAT TGC CTC CAC TGA AT-3′) and Inv Rev (5′-GCC GTG CAC TAA ACC TCG TTT GAA GAT TA-3′), treated with *DpnI* to digest methylated parental plasmids, and self-ligated. The plasmid was digested with *Apa*I and *Apa*LI and then cloned into the suicide vector pYAK1.

The generated plasmids, pYAK1-*pomA*KO and pYAK1-*cheY*KO, were introduced into *Escherichia coli* BW19795. The transformants were combined with *V. vulnificus* on a nitrocellulose Hybond C membrane (GE Healthcare, Tokyo, Japan) for conjugation, placed onto an M9 agar plate, and incubated at 25°C (Forsyth and Kushner, 1970; de Lorenzo et al., 1990; Pobigaylo et al., 2006). The bacterial suspension was plated onto TCBS agar containing 10 μg/ml chloramphenicol and incubated overnight at 37°C for selection (Pfeffer and Oliver, 2003). *V. vulnificus* CMCP6, retaining pYAK1- *pomA*KO and pYAK1-*cheY*KO, were cultured in LB broth containing 20% sucrose. The resulting strain due to *sacB*-assisted allelic exchange was named Δ*pomA* and Δ*cheY* (Miller, 1972; Kodama et al., 2002).

### Complementation of Δ*cheY*

The *cheY* gene was amplified using specific primers: *cheY* Fw (5′-GGA TCC TTG AAT AAA AAC ATG AAG ATC CTT ATT-3′) and *cheY* Rev (5′-CTC GAG TTA TAA ACG TTC AAA AAT TTT ATC TAG-3′). It cloned into pACYC184 by Infusion cloning reactions (Clontech, TaKaRa, Shiga, Japan). The complementing plasmid pACYC184-*cheY* was introduced into *V. vulnificus* via electroporation. After inoculation onto LB plate containing 10 μg/ml chloramphenicol and incubation overnight at 37°C for selection, the strain retaining the plasmid was named p*cheY*. The *cheY* gene could be expressed from the *cat* promotor on this plasmid (Wiesner et al., 2003).

### Swarming Assay

*Vibrio vulnificus* CMCP6 parent strain (WT) were grown in LB medium, and each signature-tagged transposon insertion mutants were grown in LB medium containing 100 μg/ml of kanamycin at 37°C. Overnight cultures (100 μl) were inoculated into 2 ml of fresh medium and incubated for 2 h. Log-phase bacteria were inoculated onto LB plates containing 0.3% agar and incubated for 12 h at 37°C.

### Capsule Assay

WT and E4 were grown in LB medium, and each signature-tagged transposon insertion mutants were grown in LB medium containing 100 μg/ml of kanamycin at 37°C. Overnight cultures (5 μl) were inoculated onto LB plate and incubated for 12 h at 37°C. The opacity of the colonies was examined.

### Bacterial Proliferation Analysis in Muscle Tissue

WT andΔ*cheY* were grown in LB medium containing 50 mg/ml of rifampicin, and p*cheY* was grown in LB medium containing 50 mg/ml of rifampicin and 10 μg/ml chloramphenicol with agitation (163 rpm) at 37°C. Overnight cultures (100 μl) were inoculated into 2 ml of fresh medium and incubated for 2 h. Bacteria were harvested, washed with PBS (pH 7.2) containing 0.1% gelatin, and resuspended in fresh medium. Then, 10^6^ CFU/mouse were subcutaneously inoculated into right caudal thighs. Infected mice were sacrificed at 6 h post-infection. The collected muscular tissue was suspended in PBS containing 0.1% gelatin, homogenized for 5 s with a lab mixer IKA EUROSTAR digital (IKA Werke, Germany; 1,300 rpm), and centrifuged at 800 rpm for 5 min. The supernatant was plated at 10-fold serial dilutions in duplicate on LB agar containing 50 μg/ml rifampicin and incubated for 12 h at 37°C. *V. vulnificus* colonies were counted, and the number of CFU/g of muscle tissue was determined as a bacterial burden in muscle tissue.

## Results

### Screening of Mutants in the Wound Infection Model and Identification of Crucial *V. vulnificus* Genes for Proliferation in the Healthy Hosts

**Figure 2:**
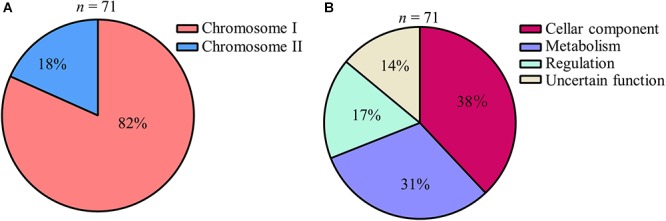
Broad grouping of genes detected by STM screening **(A,B)** Genes detected by STM were broadly grouped. Pie charts showing their chromosomal localization **(A)** and their predicted function **(B)**.

A total of 86 sets of libraries consisting of 5418 independent transposon insertion mutants were examined by comparing the presence of each tagged mutant between the input pool and the output pool samples (Figure 1). Overall, 71 mutants produced weak signals or lacked any signals upon comparing the hybridized output pool with the input pool (Figure 1), and were selected as attenuated mutant strains in which a virulence gene was disrupted by the insertion of a transposon. Although 11 out of 71 clones (5 genes: VV1_0217, VV1_0400, VV1_0778, VV1_2145, and VV2_0843) were detected twice or three times in this study, the transposon insertion sites of all attenuated strains were different even within the same gene. Of the identified genes, 82% were located in chromosome I and 18% were in chromosome II (Figure 2A). The encoded proteins of transposon-inserted genes were broadly grouped based on the KEGG pathway database^3^ and their putative function: cellular component, metabolism, regulation, and uncertain function proteins (Figure 2B).

### Flagellum and Pili

**Figure 3:**
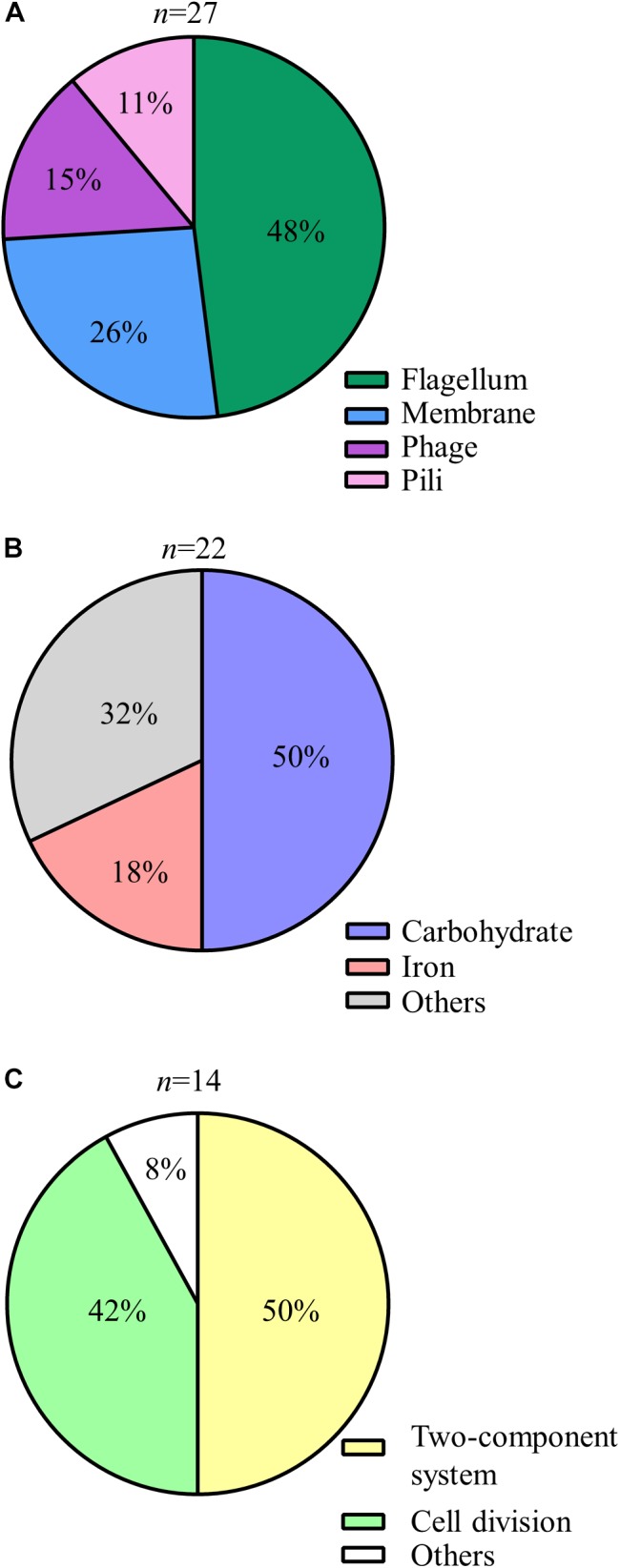
Detailed classification of the identified genes **(A–C)** Pie charts showing details of the predicted functions of encoded proteins of the genes detected by STM: cellar component **(A)**, metabolism **(B)**, and regulation **(C)**.

**Figure 4:**
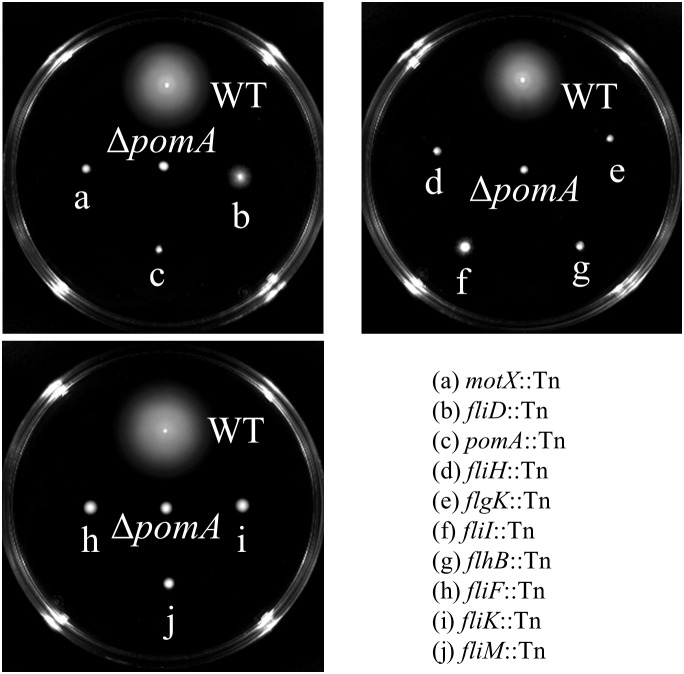
Swarming motility of WT and the mutants lacked genes involved in the flagellum.

Genes that express putative cellular component proteins were found to be involved in the construction of the flagellum, membranes, phage, and pili (Figure 3A). *V. vulnificus* has a polar single flagellum. Flagellar-based motility is involved in swimming in liquid medium and swarming on surfaces (Ottemann and Miller, 1997). A list of detected genes involved in the flagellum and its predicted function are summarized in Table 1. The detected genes were involved in the assembly or components of the flagellar MS-ring (*fliF*), C-ring (*fliM*), hook (*flgK*), filament cap (*fliD*), and motors (*pomA* and *motX*) (Yorimitsu and Homma, 2001; Ran Kim and Haeng Rhee, 2003; Evans et al., 2014; Gao et al., 2014). Genes for a component of the flagellar Type III secretion system (*fliH*, *fliI*, and *flhB*) and flagellar hook-length control (*fliK*) were responsible for flagellin export (Minamino et al., 2008). All strains carrying a disruption of any gene for flagellar proteins exhibited a defect in swarming motility (Table 1 and Figure 4). Genes for FimT, FimV, and CpaE-like proteins are involved in the synthesis of Type IV pili (Tfp) (Table 1). Our findings indicate that the flagellum and pili of *V. vulnificus* are essential for the wound infection.

**Table 1:** Genes involved in *V. vulnificus* wound infection.

Gene tag	Name	Gene product	*In vitro* phenotype	Predicted function in the wound infection
VV1_0217	*flgK*	Flagellar hook filament junction	• Non-motile	• Spread in soft-tissues
VV1_0312	*pomA*	Flagellar Na+ motor (torque generation)	• Non-motile	• Transition to the systemic
VV1_1300	*motX*	Flagellar Na+ motor component	• Non-motile	circulation
VV1_1928	*fliD*	Flagellar filament cap	• Non-motile	
VV1_1935	*fliF*	Flagellar MS-ring	• Non-motile	
VV1_1937	*fliH*	Fla export; negative regulator of FliI	• Non-motile	
VV1_1938	*fliI*	Fla export ATPase	• Non-motile	
VV1_1940	*fliK*	Flagellar hook-length control	• Non-motile	
VV1_1942	*fliM*	Flagellar motor switch component/C-ring	• Non-motile	
VV1_1948	*flhB*	Fla export	• Non-motile	
VV1_1953	*cheY*	Transmits chemoreceptor signals	• Smooth bias swimming	
VV1_1955	*cheA*	Sensor kinase	• Smooth bias swimming	
VV1_1958	*cheW*	Purine-binding chemotaxis protein	• Non-motile	

VV1_0352	*fimT*	Tfp pilus assembly		• Adhesion to cells
				• Biofilm formation


VV1_1991	*fimV*	ATPase AAA pilus assembly		
VV1_2333		Pilus assembly (CpaE-like protein)		

VV1_0578	*murG*	Glycosyltransferase		• Maintenance of bacterial cell morphology

VV1_0778		Glycosyltransferase	• Translucent colony	• Resistance to immune cells in soft tissue and the systemic circulation
VV1_1426	*wza*	Lipid A core-O-antigen ligase	• Non-motile	• Induction of inflammatory cytokines
VV1_0786	*gpsK*	Polysaccharide export	• Translucent colony	
VV1_1667		Glucosamine kinase		

VV2_0843	*vuuA*	Ferric vulnibactin receptor		• Siderophore-mediated iron acquisition
VV2_1016	*iutA*	Aerobactin siderophore receptor		

VV1_2145	*mukB*	Chromosome partition		• Chromosome partition and cell division.
VV1_2245		DNA polymerase III subunit epsilon		
VV2_0122		DNA helicase IV		

### Carbohydrate and Iron Metabolism

**Figure 5:**
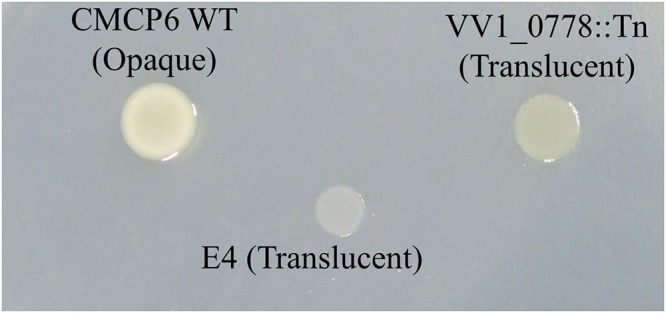
Colony morphology of WT, environmental strain E4, and VV1_0778::Tn.

The genes involved in metabolism included those responsible for the utilization of carbohydrate and iron (Figure 3B). Glycosyltransferase gene of N-acetylglucosamine (GlcNAc) metabolism was primarily detected in carbohydrate metabolism (Figure 3B and Table 1). The metabolism and biosynthesis of GlcNAc are required for the bacterial cell wall, peptidoglycans, and outer membrane; i.e., lipopolysaccharides (LPS) (Low et al., 2010; Typas et al., 2012; Lee et al., 2013). In addition, strains carrying a disruption of glycosyltransferase (VV1_0778) gene exhibited translucent colony (Figure 5), meaning reduced capsular polysaccharides (CPS) expression (Lee et al., 2013).

In iron metabolism, the genes for VuuA and IutA were detected in this study (Table 1 and Figure 3B). These are outer membrane receptors of iron chelators (siderophores) that is a high affinity protein against iron, which is necessary for bacterial proliferation (Webster and Litwin, 2000; Tanabe et al., 2005; Weinberg, 2009; Kawano et al., 2017). Thus, these findings indicate that *V. vulnificus* requires an iron acquisition system for their proliferation in healthy hosts.

### Regulation of Chemotaxis and Cell Division

Mutant strains of chromosome partition protein MukB, DNA helicase, and DNA polymerase showed equivalent proliferation as the parent strain *in vitro* but were detected by the STM method (Table 1). It indicates the existence of regulators of chromosomal replication functioning only *in vivo*. Two transposon insertion mutants that lacked chromosome partition protein MukB were detected in the STM. Each mutant was expressed 918 or 1269 amino acid residues of MukB, which is composed of 1484 amino acid residues. Both *mukB*::Tn mutants did not exhibit a growth defect *in vitro*. We have attempted to construct an in-frame deletion mutant of MukB to verify the phenotypes of *mukB*::Tn, but it could not be obtained. Thus, our results suggest that MukB is essential for the survival of *V. vulnificus* and the C-terminal domain of MukB likely plays key roles *in vivo*.

**Figure 6:**
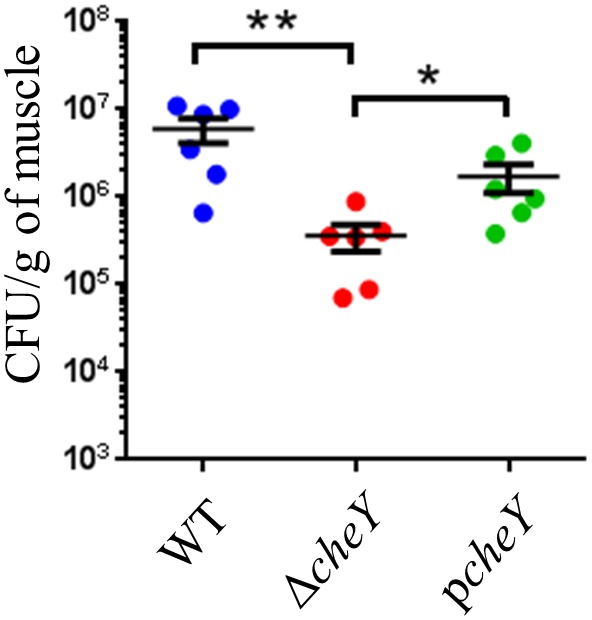
Bacterial burdens calculated as CFU/g in the muscle tissue of mice (*n* = 6/group) s.c.-inoculated with WT, Δ*cheY*, and p*cheY* at 6 h post-infection. Error bars indicate SEM. ^∗^*P* < 0.05, ^∗∗^*P* < 0.01; Mann-Whitney *U*-test.

In order to sense and respond to various environmental conditions, the two-component system, which regulates the direction of flagellar rotation based on sensing of chemoattractants or chemorepellents in bacterial chemotaxis (Butler and Camilli, 2005), along with regulation of cell division are essential in bacteria (Figure 3C). The two-component system consists of sensor kinase and response regulators and accomplishes signal transduction via their phosphorylation (Hoch, 2000; Wadhams and Armitage, 2004). In the present study, a histidine kinase (CheA), a response regulator (CheY), and an adapter protein (CheW) for chemoreceptors and the histidine kinase in *V. vulnificus* chemotaxis were detected (Table 1; Zhu et al., 2013). To determine a responsible gene for chemotaxis and whether the chemotaxis is essential for proliferation at the site of the wound infection, we inoculated WT, Δ*cheY*, or p*cheY* into the murine subcutaneous tissue and then analyzed bacterial burdens in the muscle tissue beneath the inoculation site at 6 h post-infection. Average CFU values in WT-infected mice was 5.97 × 10^6^ CFU/g. In contrast, the average CFU in the Δ*cheY*-infected mice was significantly lower, 3.59 × 10^4^ CFU/g (Figure 6). The Δ*cheY* mutant exhibits approximately 15-fold reduced bacterial burden (*p* = 0.0043) in the tissue compared with WT, and the bacterial burden was recovered by complementation with *cheY* (Figure 6). These results demonstrate that chemotaxis, as well as flagellum construction, were essential for *V. vulnificus* proliferation at the local site of the wound infection.

## Discussion

### Chromosomal Location of Functional Genes

We classified and selected 71 clones from among 5418 independent transposon insertion mutants. Although genes involved in the known virulence factors of *V. vulnificus*, such as MARTX, VVH, VvpE, the and iron acquisition system, are located on chromosome II (Miyoshi et al., 1993; Jeong et al., 2000; Chen et al., 2003; Jones and Oliver, 2009), our detected genes mainly were localized on chromosome I. These findings indicate that the genes encoding essential functions for proliferation and spreading in healthy hosts during wound infection are preferentially situated on chromosome I.

### Adhesion and Biofilm Formation

**Figure 7:**
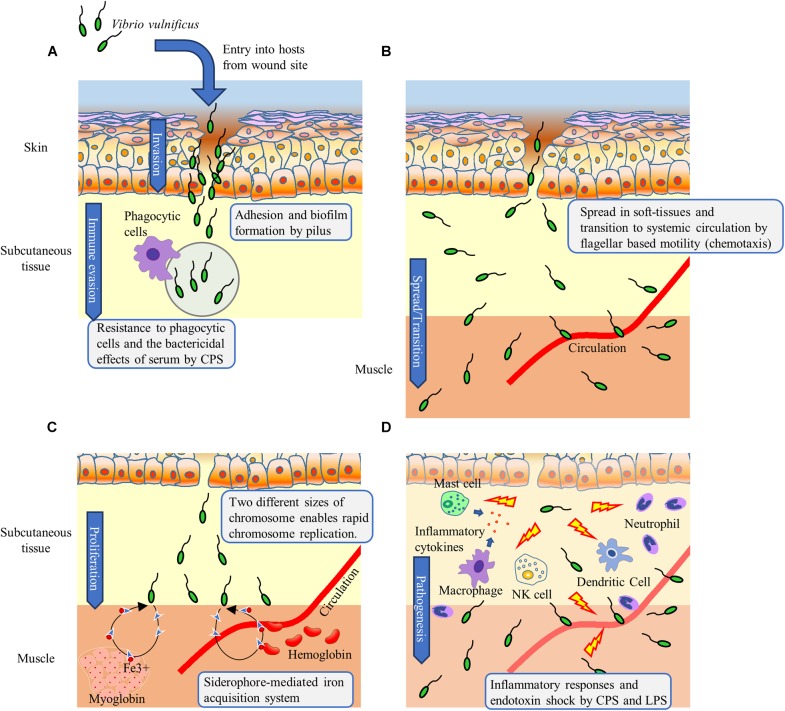
STM screening reveals the essential factors whereby *V. vulnificus* invades, spreads, proliferates, and causes wound infection. **(A)** *V. vulnificus* adheres to cells located at the local wound infection site via the pilus. Biofilm formation and CPS contribute to resistance against immune cells. **(B)** *V. vulnificus* spreads in soft-tissues by flagellar based motility based on chemotaxis. **(C)** Two different sizes of chromosomes enable rapid chromosome replication leading to rapid cell division and proliferation. Iron, which is an essential element in bacterial metabolic and informational cellular pathways, is acquired via a siderophore-mediated system. **(D)** CPS and LPS induce inflammation-associated cytokines leading to septic shock.

Type IV pili are known to play a role in cell adhesion, twitching motility, and the uptake of foreign genes (Paranjpye and Strom, 2005; Bucior et al., 2012). Studies on *Pseudomonas aeruginosa* indicate that the FimT, FimV, and CpaE-like protein are involved in Tfp assembly (Michel et al., 2011). Tfp of *P. aeruginosa* is essential for twitching motility, however, that of *V. vulnificus* is expected to be functionally distinct (Jones and Oliver, 2009; Bucior et al., 2012). Pili contribute to pathogenicity via biofilm formation and adhesion to epithelial cells in *V. vulnificus* infection (Paranjpye et al., 1998; Paranjpye and Strom, 2005; Jones and Oliver, 2009). Gander and LaRocco (1989) showed that clinically isolated strains of *V. vulnificus*, especially those isolated from wound infection sites, presented higher numbers of pilus fibers per cell than environmentally isolated strains. Paranjpye and Strom (2005) further reported that pili-deficient mutants of *V. vulnificus* exhibited decreased adhesion to cells, biofilm formation, and lethality in iron dextran-treated mice. Together with these data, the results of the present study first showed the essential genes for and importance of pili in the wound infection, suggesting the possibility that Tfp of *V. vulnificus* may be involved in the adherence to cells and biofilm formation in soft tissues at the wound infection site (Figure 7A).

### Bacterial Spreading in Soft-Tissues and Transition to the Systemic Circulation

The flagellum is structurally composed of the basal body, hook, and filament (Ran Kim and Haeng Rhee, 2003; Hirano et al., 2009; Evans et al., 2014; Gao et al., 2014; Tsang and Hoover, 2015). The basal body serves as a rotation axis and consists of the MS-ring, C-ring, PL-ring, and rod. The C-ring of the basal body interacts with the motor protein and functions as a rotor (Yorimitsu and Homma, 2001). The hook transmits the torque generated in the basal body to the filament. The basal body rod, hook, and filament are transported through the central channel of the flagellum by the flagellar Type III secretion system that exists in the cytoplasm (Minamino et al., 2008; Parker et al., 2014). The filament is made of polymerized flagellin proteins and forms a spiral shape. It has been reported that mutants disrupting flagellar hook-basal body, flagellin, and controlling flagellum expression exhibited significant decreases in invasion ability from the intestine into the systemic circulation, as well as in lethality during intragastric infection of suckling mice (Kim et al., 2003, 2014; Duong-Nu et al., 2016). In addition, we reported that immunization with flagellin can prevent *V. vulnificus* proliferation in a local wound infection site (Yamazaki et al., 2017). These findings show that the flagellum is required for proliferation both in the intestine and in soft-tissues at the local wound infection site and for invasion of the systemic circulation (Figure 7B).

Bacteria change swimming direction by tumbling consequent to switching the direction of flagellar rotation from counterclockwise to clockwise (Homma et al., 1996; Zhu et al., 2013) and move toward a favorable environment through the proper control of flagellar rotation based on chemotaxis, which is controlled by the two-component system (Butler and Camilli, 2005). Methyl-accepting chemosensory proteins (MCPs) sense chemical gradients of the surrounding environments, bind chemoattractants or chemorepellents, and transmit chemotactic signals to CheW interacting with CheA (Zhu et al., 2013). CheA is involved in the activation (phosphorylation) of CheY. When the activated CheY binds to the switch protein FliM, the direction of flagellar rotation is altered from counterclockwise to clockwise (Delalez et al., 2010; Biswas et al., 2013). Our observation that CheY and CheA mutants swim straight is a consequence of counterclockwise-biased flagellar rotation (smooth biased) (Table 1). They will swim straight *in vivo* as well as *in vitro* and may not able to spread in soft tissues which have complex and intricate structures. It must have resulted in a non-sufficient proliferation by Δ*cheY* at the local wound infection site and not be detected from spleen as a STM output (Figure 6). Together, our findings revealed that in addition to the complete flagellum, flagellar rotation control based on chemotaxis is essential for *V. vulnificus* to spread in soft tissue and proliferate at the site of wound infection. Notably, the chemotaxis of *V. vulnificus* associated with tissue tropism or invasion of the systemic circulation has not previously been investigated. However, although approximately 40 genes encoding MCPs exist on *V. vulnificus* chromosomes, these were not detected in the present study. Identification of the chemoattractants underlying bacterial spread in soft tissue and transition to the systemic circulation will therefore likely be helpful to prevent the development of sepsis.

### Rapid Proliferation

Several pathogens, such as *Salmonella enterica serovar* Typhi, *Vibrio cholerae*, and *E. coli*, secrete siderophore during intestinal tract infection (Weinberg, 2009). It is thought that *V. vulnificus* also secretes siderophore in primary septicemia through the digestion of raw seafood (Simpson and Oliver, 1983; Litwin and Byrne, 1998; Jones and Oliver, 2009; Tan et al., 2014; Oliver, 2015). Notably, patients with primary septicemia have underlying diseases such as liver cirrhosis, hemochromatosis, or alcoholism, which predispose to iron overload. In fact, the elevation of serum iron levels in hosts is highly associated with *V. vulnificus* infection (Biosca et al., 1996; Litwin et al., 1996; Arezes et al., 2015). Unlike in such patients, iron is limited in a healthy host and usually stored as heme in erythrocytes (hemoglobin) and muscle (myoglobin), or ferritin in hepatocytes. Extracellular iron is bound to transferrin in the serum and lactoferrin in secretions. In addition, Arezes et al. (2015) reported that the hepcidin secreted by hepatocytes suppresses serum iron levels during *V. vulnificus* infection. Unlike in septicemia, in soft-tissue infection, one a single study has reported that *P. aeruginosa* secretes siderophore, in a murine model of surgical site infection (Kim et al., 2015). These observations indicate that it would be difficult for pathogens to acquire iron from the iron regulatory system in a healthy host. Our result is strongly suggested that siderophore secretion and siderophore receptors (ferric vulnibactin receptor and aerobactin siderophore receptor) are essential to acquire iron for the proliferation of *V. vulnificus* during wound infection in healthy hosts (Figure 7C). It is consistent with the previous report that the expression of these receptors in *V. vulnificus* is enhanced under *in vitro* iron-restricted conditions (Kawano et al., 2017).

*Vibrionaceae* bacteria such as *V. vulnificus*, *V. cholerae*, and *Vibrio parahaemolyticus* have two differently sized chromosomes that each contain an origin for replication (Sawitzke and Austin, 2000; Chen et al., 2003; Rasmussen et al., 2007; Val et al., 2016). This characteristic enables rapid chromosome replication, cell division, and proliferation and is likely essential for the proliferation of *V. vulnificus* in the wound infection. In particular, several studies on *E. coli* have shown that MukB acts as a homodimer, condenses DNA, and facilitates chromosome segregation (Niki et al., 1992).

We also detected a *murG* mutant by STM. In *P. aeruginosa* and *E. coli*, MurG plays a key role in the biosynthesis of the peptidoglycan layer to form a glycosidic bond between N-acetylmuramyl pentapeptide and GlcNAc for bacterial cell walls (Mengin-Lecreulx et al., 1991; Brown et al., 2013; Dhar et al., 2017; Saxena et al., 2017). The bacterial cell wall contributes to cellular integrity, determination of shape, and adaptation to the surrounding environment, allowing cell expansion during growth and cell separation after cell division. Accordingly, mutation in the cell wall composition affected not only morphology of the bacterial cells but also the rapid cell division (Figure 7C).

### Immune Evasion and Pathogenesis

Park et al. (2006) and Senchenkova et al. (2009) showed a mutant strain of *wbpP* gene encoding GlcNAc C4 epimerase is CPS-deficient. Thus, GlcNAc metabolism is essential for the synthesis of CPS. CPS is considered essential for *in vivo* proliferation, as clinically isolated strains typically produce CPS unlike environmentally isolated strains (Yoshida et al., 1985; Simpson et al., 1987). It has been reported that CPS of *V. vulnificus* is essential for the evasion of phagocytosis by macrophages as well as for serum resistance (Wright et al., 2001; Williams et al., 2014). In addition, capsular (opaque) strains are more invasive than non-capsular (translucent) strain in guinea pig subcutaneous tissue (Yoshida et al., 1985; Figure 7A). Synthesized CPS is transported through a channel in the bacterial outer membrane formed by the lipoprotein Wza, of which deficient mutants showed translucent colonies, increased serum sensitivity, and reduced lethality in mice (Wright et al., 2001). In turn, LPS constitutes a polysaccharide side chain that comprises lipid A, core oligosaccharide, and O antigen. The lipid A core-O-antigen ligase that was detected in the present study functions to bind O-antigen polysaccharide to the lipid A-core oligosaccharide (Abeyrathne et al., 2005). It was reported that sialic acid modification of LPS is required for *V. vulnificus* to proliferate in the systemic circulation (Lubin et al., 2015). However, Wza and the sialic acid modification of LPS are also involve in flagellar assembly (Lubin et al., 2015). These results complicate the interpretation of which factor(s) among CPS, LPS, or the flagellum is the most important for each function. However, although further study is required to clarify this issue, the importance of CPS, LPS, and the flagellum was suggested by the detection of mutants that specifically disrupted each factor in our STM (Table 1). In addition, CPS and LPS of *V. vulnificus* serve as inducers of tumor necrosis factor-alpha (Powell et al., 1997), which has the potential to cause endotoxic shock. Together, our findings suggest that LPS and CPS of *V. vulnificus* contribute to the initial establishment and fatal outcomes in both primary septicemia and wound infection (Figures 7A,D).

## Conclusion

Our findings demonstrate that the pathogenicity of *V. vulnificus* during wound infection highly depends on its characteristics; motility, iron-acquisition, CPS, LPS, and two circular chromosomes. In addition, we identified novel factor: chemotaxis, which likely enables *V. vulnificus* to spread toward a suitable environment, rapidly proliferate especially at the local infection site, and aggravate wound infection within a short period. These factors comprise attractive potential candidates for the development of antibiotics or vaccines to control infection.

## Author Contributions

KY and TKas contributed to the conception and design of the study. KY performed the majority of the experiments and analyzed the data. MM and TKad assisted STM. KY wrote the manuscript. SU analyzed and supervised this study. All authors contributed to manuscript revision and read and approved the submitted version.

## Conflict of Interest Statement

The authors declare that the research was conducted in the absence of any commercial or financial relationships that could be construed as a potential conflict of interest.

## Footnotes

1 http://www.jgi.doe.gov/programs/GEBA/index.html

2 https://www.genome.jp/kegg

3 https://www.genome.jp/kegg/pathway.html

## References

[B1] Abeyrathne P. D. Daniels C. Poon K. K. Matewish M. J. Lam J. S. (2005). Functional characterization of WaaL, a ligase associated with linking O-antigen polysaccharide to the core of *Pseudomonas aeruginosa* lipopolysaccharide. *J. Bacteriol.* 187 3002–3012. 10.1128/JB.187.9.3002-3012.2005 15838026 PMC1082828

[B2] Arezes J. Jung G. Gabayan V. Valore E. Ruchala P. Gulig P. A. (2015). Hepcidin-induced hypoferremia is a critical host defense mechanism against the siderophilic bacterium *Vibrio vulnificus*. *Cell Host Microbe* 17 47–57. 10.1016/j.chom.2014.12.001 25590758 PMC4296238

[B3] Biosca E. G. Fouz B. Alcaide E. Amaro C. (1996). Siderophore-mediated iron acquisition mechanisms in *Vibrio vulnificus* biotype 2. *Appl. Environ. Microbiol.* 62 928–935. 8975620 10.1128/aem.62.3.928-935.1996 PMC167857

[B4] Biswas M. Dey S. Khamrui S. Sen U. Dasgupta J. (2013). Conformational barrier of CheY3 and inability of CheY4 to bind FliM control the flagellar motor action in *Vibrio cholerae*. *PLoS One* 8:e73923. 10.1371/journal.pone.0073923 24066084 PMC3774744

[B5] Brown K. Vial S. C. Dedi N. Westcott J. Scally S. Bugg T. D. (2013). Crystal structure of the *Pseudomonas aeruginosa* MurG: UDP-GlcNAc substrate complex. *Protein Pept. Lett.* 20 1002–1008. 10.2174/0929866511320090006 22973843

[B6] Bucior I. Pielage J. F. Engel J. N. (2012). *Pseudomonas aeruginosa* pili and flagella mediate distinct binding and signaling events at the apical and basolateral surface of airway epithelium. *PLoS Pathog.* 8:e1002616. 10.1371/journal.ppat.1002616 22496644 PMC3320588

[B7] Butler S. M. Camilli A. (2005). Going against the grain: chemotaxis and infection in *Vibrio cholerae*. *Nat. Rev. Microbiol.* 3 611–620. 10.1038/nrmicro1207 16012515 PMC2799996

[B8] Chen C. Y. Wu K. M. Chang Y. C. Chang C. H. Tsai H. C. Liao T. L. (2003). Comparative genome analysis of *Vibrio vulnificus*, a marine pathogen. *Genome Res.* 13 2577–2587. 10.1101/gr.1295503 14656965 PMC403799

[B9] Chung K. J. Cho E. J. Kim M. K. Kim Y. R. Kim S. H. Yang H. Y. (2010). RtxA1-Induced expression of the small GTPase Rac2 plays a key role in the pathogenicity of *Vibrio vulnificus*. *J. Infect. Dis.* 201 97–105. 10.1086/648612 19919301

[B10] de Lorenzo V. Herrero M. Jakubzik U. Timmis K. N. (1990). Mini-Tn5 transposon derivatives for insertion mutagenesis, promoter probing, and chromosomal insertion of cloned DNA in gram-negative eubacteria. *J. Bacteriol.* 172 6568–6572. 10.1128/jb.172.11.6568-6572.1990 2172217 PMC526846

[B11] Delalez N. J. Wadhams G. H. Rosser G. Xue Q. Brown M. T. Dobbie I. M. (2010). Signal-dependent turnover of the bacterial flagellar switch protein FliM. *Proc. Natl. Acad. Sci. U.S.A.* 107 11347–11351. 10.1073/pnas.1000284107 20498085 PMC2895113

[B12] Dhar S. Kumari H. Balasubramanian D. Mathee K. (2017). Cell-wall recycling and synthesis in *Escherichia coli* and *Pseudomonas aeruginosa* – their role in the development of resistance. *J. Med. Microbiol.* 67 1–21. 10.1099/jmm.0.000636 29185941

[B13] Duong-Nu T. M. Jeong K. Hong S. H. Nguyen H. V. Ngo V. H. Min J. J. (2016). All three TonB systems are required for *Vibrio vulnificus* CMCP6 tissue invasiveness by controlling flagellum expression. *Infect. Immun.* 84 254–265. 10.1128/IAI.00821-15 26527216 PMC4693995

[B14] Evans L. D. Hughes C. Fraser G. M. (2014). Building a flagellum outside the bacterial cell. *Trends Microbiol.* 22 566–572. 10.1016/j.tim.2014.05.009 24973293 PMC4183434

[B15] Forsyth M. P. Kushner D. J. (1970). Nutrition and distribution of salt response in populations of moderately halophilic bacteria. *Can. J. Microbiol.* 16 253–261. 10.1139/m70-047 5429473

[B16] Gander R. M. LaRocco M. T. (1989). Detection of piluslike structures on clinical and environmental isolates of *Vibrio vulnificus*. *J. Clin. Microbiol.* 27 1015–1021. 2568368 10.1128/jcm.27.5.1015-1021.1989 PMC267474

[B17] Gao B. Lara-Tejero M. Lefebre M. Goodman A. L. Galán J. E. (2014). Novel components of the flagellar system in epsilonproteobacteria. *mBio* 5:e01349-14. 10.1128/mBio.01349-14 24961693 PMC4073491

[B18] Gulig P. A. Tucker M. S. Thiaville P. C. Joseph J. L. Brown R. N. (2009). USER friendly cloning coupled with chitin-based natural transformation enables rapid mutagenesis of *Vibrio vulnificus*. *Appl. Environ. Microbiol.* 75 4936–4949. 10.1128/AEM.02564-08 19502446 PMC2725515

[B19] Hensel M. Shea J. E. Gleeson C. Jones M. D. Dalton E. Holden D. W. (1995). Simultaneous identification of bacterial virulence genes by negative selection. *Science* 269 400–403. 10.1126/science.7618105 7618105

[B20] Hirano T. Mizuno S. Aizawa S. I. Hughes K. T. (2009). Mutations in *flk*, *flgG*, *flhA*, and *flhE* that affect the flagellar type III secretion specificity switch in *Salmonella enterica*. *J. Bacteriol.* 191 3938–3949. 10.1128/JB.01811-08 19376867 PMC2698386

[B21] Hoch J. A. (2000). Two-component and phosphorelay signal transduction. *Curr. Opin. Microbiol.* 3 165–170. 10.1016/S1369-5274(00)00070-9 10745001

[B22] Homma M. Oota H. Kojima S. Kawagishi I. Imae Y. (1996). Chemotactic responses to an attractant and a repellent by the polar and lateral flagellar systems of *Vibrio alginolyticus*. *Microbiology* 142 2777–2783. 10.1099/13500872-142-10-2777 8885393

[B23] Jeong H. G. Satchell K. J. (2012). Additive function of *Vibrio vulnificus* MARTXVv and VvhA cytolysins promotes rapid growth and epithelial tissue necrosis during intestinal infection. *PLoS Pathog.* 8:e1002581. 10.1371/journal.ppat.1002581 22457618 PMC3310748

[B24] Jeong K. C. Jeong H. S. Rhee J. H. Lee S. E. Chung S. S. Starks A. M. (2000). Construction and phenotypic evaluation of a *Vibrio vulnificus* vvpE mutant for elastolytic protease. *Infect. Immun.* 68 5096–5106. 10.1128/IAI.68.9.5096-5106.2000 10948131 PMC101747

[B25] Jones M. K. Oliver J. D. (2009). *Vibrio vulnificus*: disease and pathogenesis. *Infect. Immun.* 77 1723–1733. 10.1128/IAI.01046-08 19255188 PMC2681776

[B26] Kashimoto T. Iwasaki C. Gojo M. Sugiyama H. Yoshioka K. Yamamoto Y. (2015). *Vibrio vulnificus* detected in the spleen leads to fatal outcome in a mouse oral infection model. *FEMS Microbiol. Lett.* 362:fnv0005. 10.1093/femsle/fnv005 25790509

[B27] Kashimoto T. Ueno S. Hanajima M. Hayashi H. Akeda Y. Miyoshi S. (2003). *Vibrio vulnificus* induces macrophage apoptosis in vitro and in vivo. *Infect. Immun.* 71 533–535. 10.1128/IAI.71.1.533-535.2003 12496206 PMC143416

[B28] Kashimoto T. Ueno S. Hayashi H. Hanajima M. Yoshioka K. Yoshida K. (2005). Depletion of lymphocytes, but not neutrophils, via apoptosis in a murine model of *Vibrio vulnificus* infection. *J. Med. Microbiol.* 54 15–22. 10.1099/jmm.0.45861-0 15591250

[B29] Kawano H. Miyamoto K. Negoro M. Zushi E. Tsuchiya T. Tanabe T. (2017). IutB participates in the ferric-vulnibactin utilization system in *Vibrio vulnificus* M2799. *Biometals* 30 203–216. 10.1007/s10534-017-9994-0 28150143

[B30] Kim M. Christley S. Khodarev N. N. Fleming I. Huang Y. Chang E. (2015). *Pseudomonas aeruginosa* wound infection involves activation of its iron acquisition system in response to fascial contact. *J. Trauma Acute Care Surg.* 78 823–829. 10.1097/TA.0000000000000574 25807409 PMC4376013

[B31] Kim S. Y. Thanh X. T. Jeong K. Kim S. B. Pan S. O. Jung C. H. (2014). Contribution of six flagellin genes to the flagellum biogenesis of *Vibrio vulnificus* and in vivo invasion. *Infect. Immun.* 82 29–42. 10.1128/IAI.00654-13 24101693 PMC3911872

[B32] Kim Y. R. Lee S. E. Kim C. M. Kim S. Y. Shin E. K. Shin D. H. (2003). Characterization and pathogenic significance of *Vibrio vulnificus* antigens preferentially expressed in septicemic patients. *Infect. Immun.* 71 5461–5471. 10.1128/IAI.71.10.5461-5471.2003 14500463 PMC201039

[B33] Kim Y. R. Lee S. E. Kook H. Yeom J. A. Na H. S. Kim S. Y. (2008). *Vibrio vulnificus* RTX toxin kills host cells only after contact of the bacteria with host cells. *Cell. Microbiol.* 10 848–862. 10.1111/j.1462-5822.2007.01088.x 18005241

[B34] Kodama T. Akeda Y. Kono G. Takahashi A. Imura K. Iida T. (2002). The EspB protein of enterohaemorrhagic *Escherichia coli* interacts directly with α-catenin. *Cell. Microbiol.* 4 213–222. 10.1046/j.1462-5822.2002.00176.x 11952638

[B35] Lee K. E. Bang J. S. Baek C. H. Park D. K. Hwang W. Choi S. H. (2007). IVET-based identification of virulence factors in *Vibrio vulnificus* MO6-24/O. *J. Microbiol. Biotechnol.* 17 234–243. 18051754

[B36] Lee K. J. Kim J. A. Hwang W. Park S. J. Lee K. H. (2013). Role of capsular polysaccharide (CPS) in biofilm formation and regulation of CPS production by quorum-sensing in *Vibrio vulnificus*. *Mol. Microbiol.* 90 841–857. 10.1111/mmi.12401 24102883

[B37] Lin T. Troy E. B. Hu L. T. Gao L. Norris S. J. (2014). Transposon mutagenesis as an approach to improved understanding of *Borrelia* pathogenesis and biology. *Front. Cell. Infect. Microbiol.* 4:63. 10.3389/fcimb.2014.00063 24904839 PMC4033020

[B38] Litwin C. M. Byrne B. L. (1998). Cloning and characterization of an outer membrane protein of *Vibrio vulnificus* required for heme utilization: regulation of expression and determination of the gene sequence. *Infect. Immun.* 66 3134–3141. 9632577 10.1128/iai.66.7.3134-3141.1998 PMC108324

[B39] Litwin C. M. Rayback T. W. Skinner J. (1996). Role of catechol siderophore synthesis in *Vibrio vulnificus* virulence. *Infect. Immun.* 64 2834–2838. 8698519 10.1128/iai.64.7.2834-2838.1996 PMC174150

[B40] Lo H. R. Lin J. H. Chen Y. H. Chen C. L. Shao C. P. Lai Y. C. (2011). RTX toxin enhances the survival of *Vibrio vulnificus* during infection by protecting the organism from phagocytosis. *J. Infect. Dis.* 203 1866–1874. 10.1093/infdis/jir070 21422475

[B41] Low D. H. P. Frecer V. Saux A. L. Srinivasan G. A. Ho B. Chen J. (2010). Molecular interfaces of the galactose-binding protein tectonin domains in host-pathogen interaction. *J. Biol. Chem.* 285 9898–9907. 10.1074/jbc.M109.059774 20118243 PMC2843237

[B42] Lubin J. B. Lewis W. G. Gilbert N. M. Weimer C. M. Almagro-Moreno S. Boyd E. F. (2015). Host-like carbohydrates promote bloodstream survival of *Vibrio vulnificus* in vivo. *Infect. Immun.* 83 3126–3136. 10.1128/IAI.00345-15 26015477 PMC4496609

[B43] Mahan M. J. Slauch J. M. Mekalanos J. J. (1993). Selection of bacterial virulence genes that are specifically induced in host tissues. *Science* 259 686–688. 10.1126/science.8430319 8430319

[B44] Mengin-Lecreulx D. Texier L. Rousseau M. van Heijenoort J. (1991). The murG gene of *Escherichia coli* codes for the UDP-N-acetylglucosamine: N-acetylmuramyl-(pentapeptide) pyrophosphoryl-undecaprenol N-acetylglucosamine transferase involved in the membrane steps of peptidoglycan synthesis. *J. Bacteriol.* 173 4625–4636. 10.1128/jb.173.15.4625-4636.1991 1649817 PMC208138

[B45] Menon M. P. Yu P. A. Iwamoto M. Painter J. (2014). Pre-existing medical conditions associated with *Vibrio vulnificus* septicaemia. *Epidemiol. Infect.* 142 878–881. 10.1017/S0950268813001593 23842472 PMC4598054

[B46] Michel G. P. Aguzzi A. Ball G. Soscia C. Bleves S. Voulhoux R. (2011). Role of fimV in type II secretion system-dependent protein secretion of *Pseudomonas aeruginosa* on solid medium. *Microbiology* 157 1945–1954. 10.1099/mic.0.045849-0 21527471

[B47] Miller J. H. (1972). *Experiments in Molecular Genetics, Cold Spring Harbor Laboratory*. New York, NY: Cold Spring Harbor.

[B48] Minamino T. Imada K. Namba K. (2008). Mechanisms of type III protein export for bacterial flagellar assembly. *Mol. Biosyst.* 4 1105–1115. 10.1039/b808065H 18931786

[B49] Miyoshi S. Oh E. G. Hirata K. Shinoda S. (1993). Exocellulr toxic factors prowced by *Vibrio vulnificus*. *J. Toxicol. Toxin Rev.* 12 253–288. 10.3109/15569549309014409

[B50] Niki H. Imamura R. Kitaoka M. Yamanaka K. Ogura T. Hiraga S. (1992). E. coli MukB protein involved in chromosome partition forms a homodimer with a rod-and-hinge structure having DNA binding and ATP/GTP binding activities. *EMBO J.* 11 5101–5109. 10.1002/j.1460-2075.1992.tb05617.x 1464330 PMC556988

[B51] Oliver J. D. (2005). Wound infections caused by *Vibrio vulnificus* and other marine bacteria. *Epidemiol. Infect.* 133 383–391. 10.1017/S0950268805003894 15962544 PMC2870261

[B52] Oliver J. D. (2015). The biology of *Vibrio vulnificus*. *Microbiol. Spectr.* 3 349–366. 10.1128/microbiolspec.VE-0001-2014 26185084

[B53] Ottemann K. M. Miller J. F. (1997). Roles for motility in bacterial–host interactions. *Mol. Microbiol.* 24 1109–1117. 10.1046/j.1365-2958.1997.4281787.x 9218761

[B54] Paranjpye R. N. Lara J. C. Pepe J. C. Pepe C. M. Strom M. S. (1998). The type IV leader peptidase/N-methyltransferase of *Vibrio vulnificus* controls factors required for adherence to HEp-2 cells and virulence in iron-overloaded mice. *Infect. Immun.* 66 5659–5668. 9826339 10.1128/iai.66.12.5659-5668.1998 PMC108715

[B55] Paranjpye R. N. Strom M. S. (2005). A *Vibrio vulnificus* type IV pilin contributes to biofilm formation, adherence to epithelial cells, and virulence. *Infect. Immun.* 73 1411–1422. 10.1128/IAI.73.3.1411-1422.2005 15731039 PMC1064924

[B56] Park N. Y. Lee J. H. Kim M. W. Jeong H. G. Lee B. C. Kim T. S. (2006). Identification of the *Vibrio vulnificus* wbpP gene and evaluation of its role in virulence. *Infect. Immun.* 74 721–728. 10.1128/IAI.74.1.721-728.2006 16369029 PMC1346593

[B57] Parker J. L. Lowry R. C. Couto N. A. Wright P. C. Stafford G. P. Shaw J. G. (2014). Maf-dependent bacterial flagellin glycosylation occurs before chaperone binding and flagellar T3SS export. *Mol. Microbiol.* 92 258–272. 10.1111/mmi.12549 24527847 PMC4065374

[B58] Pfeffer C. Oliver J. D. (2003). A comparison of thiosulphate-citrate-bile salts-sucrose (TCBS) agar and thiosulphate-chloride-iodide (TCI) agar for the isolation of *Vibrio* species from estuarine environments. *Lett. Appl. Microbiol.* 36 150–151. 10.1046/j.1472-765X.2003.01280.x 12581373

[B59] Pobigaylo N. Wetter D. Szymczak S. Schiller U. Kurtz S. Meyer F. (2006). Construction of a large signature-tagged mini-Tn5 transposon library and its application to mutagenesis of *Sinorhizobium meliloti*. *Appl. Environ. Microbiol.* 72 4329–4337. 10.1128/AEM.03072-05 16751548 PMC1489598

[B60] Powell J. L. Wright A. C. Wasserman S. S. Hone D. M. Morris J. G. Jr. (1997). Release of tumor necrosis factor alpha in response to *Vibrio vulnificus* capsular polysaccharide in in vivo and in vitro models. *Infect. Immun.* 65 3713–3718. 9284142 10.1128/iai.65.9.3713-3718.1997 PMC175529

[B61] Ran Kim Y. Haeng Rhee J. (2003). Flagellar basal body flg operon as a virulence determinant of *Vibrio vulnificus*. *Biochem. Biophys. Res. Commun.* 304 405–410. 10.1016/S0006-291X(03)00613-2 12711330

[B62] Rasmussen T. Jensen R. B. Skovgaard O. (2007). The two chromosomes of *Vibrio cholerae* are initiated at different time points in the cell cycle. *EMBO J.* 26 3124–3131. 10.1038/sj.emboj.7601747 17557077 PMC1914095

[B63] Sawitzke J. A. Austin S. (2000). Suppression of chromosome segregation defects of *Escherichia coli* muk mutants by mutations in topoisomerase I. *Proc. Natl. Acad. Sci. U.S.A.* 97 1671–1676. 10.1073/pnas.030528397 10660686 PMC26494

[B64] Saxena S. Abdullah M. Sriram D. Guruprasad L. (2017). Discovery of novel inhibitors of *Mycobacterium tuberculosis* MurG: homology modeling, structure based pharmacophore, molecular docking, and molecular dynamics simulations. *J. Biomol. Struct. Dyn.* 17 1–15. 10.1080/07391102.2017.1384398 28948866

[B65] Senchenkova S. N. Shashkov A. S. Knirel Y. A. Esteve C. Alcaide E. Merino S. (2009). Structure of a polysaccharide from the lipopolysaccharide of *Vibrio vulnificus* clinical isolate YJ016 containing 2-acetimidoylamino-2-deoxy-l-galacturonic acid. *Carbohydr. Res.* 344 1009–1013. 10.1016/j.carres.2009.03.021 19368903

[B66] Simpson L. M. Oliver J. D. (1983). Siderophore production by *Vibrio vulnificus*. *Infect. Immun.* 41 644–649. 6223882 10.1128/iai.41.2.644-649.1983 PMC264691

[B67] Simpson L. M. White V. K. Zane S. F. Oliver J. D. (1987). Correlation between virulence and colony morphology in *Vibrio vulnificus*. *Infect. Immun.* 55 269–272. 2432016 10.1128/iai.55.1.269-272.1987 PMC260315

[B68] Tan W. Verma V. Jeong K. Kim S. Y. Jung C. H. Lee S. E. (2014). Molecular characterization of vulnibactin biosynthesis in *Vibrio vulnificus* indicates the existence of an alternative siderophore. *Front. Microbiol.* 5:1. 10.3389/fmicb.2014.00001 24478763 PMC3900857

[B69] Tanabe T. Naka A. Aso H. Nakao H. Narimatsu S. Inoue Y. (2005). A novel aerobactin utilization cluster in *Vibrio vulnificus* with a gene involved in the transcription regulation of the iutA homologue. *Microbiol. Immunol.* 49 823–834. 10.1111/j.1348-0421.2005.tb03671.x 16172537

[B70] Tsang J. Hoover T. R. (2015). Basal body structures differentially affect transcription of RpoN- and FliA-dependent flagellar genes in *Helicobacter pylori*. *J. Bacteriol.* 197 1921–1930. 10.1128/JB.02533-14 25825427 PMC4420910

[B71] Typas A. Banzhaf M. Gross C. A. Vollmer W. (2012). From the regulation of peptidoglycan synthesis to bacterial growth and morphology. *Nat. Rev. Microbiol.* 10 123–136. 10.1038/nrmicro2677 22203377 PMC5433867

[B72] Val M.-E. Marbouty M. de Lemos Martins F. Kennedy S. P. Kemble H. Bland M. J. (2016). A checkpoint control orchestrates the replication of the two chromosomes of *Vibrio cholerae*. *Sci. Adv.* 2:e1501914. 10.1126/sciadv.1501914 27152358 PMC4846446

[B73] Vezzulli L. Colwell R. R. Pruzzo C. (2013). Ocean warming and spread of pathogenic *Vibrios* in the aquatic environment. *Microb. Ecol.* 65 817–825. 10.1007/s00248-012-0163-2 23280498

[B74] Wadhams G. H. Armitage J. P. (2004). Making sense of it all: bacterial chemotaxis. *Nat. Rev. Mol. Cell Biol.* 5 1024–1037. 10.1038/nrm1524 15573139

[B75] Webster A. C. Litwin C. M. (2000). Cloning and characterization of vuuA, a gene encoding the *Vibrio vulnificus* ferric vulnibactin receptor. *Infect. Immun.* 68 526–534. 10.1128/IAI.68.2.526-534.2000 10639413 PMC97172

[B76] Weinberg E. D. (2009). Iron availability and infection. *Biochim. Biophys. Acta* 1790 600–605. 10.1016/j.bbagen.2008.07.002 18675317

[B77] Wiesner R. S. Hendrixson D. R. DiRita V. J. (2003). Natural transformation of *Campylobacter jejuni* requires components of a type II secretion system. *J. Bacteriol.* 185 5408–5418. 10.1128/JB.185.18.5408-5418.2003 12949093 PMC193740

[B78] Williams T. C. Ayrapetyan M. Ryan H. Oliver J. D. (2014). Serum survival of *Vibrio vulnificus*: role of genotype, capsule, complement, clinical origin, and in situ incubation. *Pathogens* 3 822–832. 10.3390/pathogens3040822 25436506 PMC4282887

[B79] Wright A. C. Powell J. L. Kaper J. B. Morris J. G. Jr. (2001). Identification of a group 1-like capsular polysaccharide operon for *Vibrio vulnificus*. *Infect. Immun.* 69 6893–6901. 10.1128/IAI.69.11.6893-6901.2001 11598064 PMC100069

[B80] Yamamoto M. Kashimoto T. Tong P. Xiao J. Sugiyama M. Inoue M. (2015). Signature-tagged mutagenesis of *Vibrio vulnificus*. *J. Vet. Med. Sci.* 77 823–828. 10.1292/jvms.14-0655 25755021 PMC4527504

[B81] Yamazaki K. Kashimoto T. Hashimoto Y. Kado T. Ueno S. (2017). Immunogenicity and protective efficacy of *Vibrio vulnificus* flagellin protein FlaB in a wound infection model. *J. Vet. Med. Sci.* 80 55–58. 10.1292/jvms.17-0395 29142160 PMC5797859

[B82] Yorimitsu T. Homma M. (2001). Na^+^-driven flagellar motor of Vibrio. *Biochim. Biophys. Acta* 1505 82–93. 10.1016/S0005-2728(00)00279-6 11248191

[B83] Yoshida S. Ogawa M. Mizuguchi Y. (1985). Relation of capsular materials and colony opacity to virulence of *Vibrio vulnificus*. *Infect. Immun.* 47 446–451. 2578434 10.1128/iai.47.2.446-451.1985 PMC263190

[B84] Zhu S. Kojima S. Homma M. (2013). Structure, gene regulation and environmental response of flagella in *Vibrio*. *Front. Microbiol.* 4:410 10.3389/fmicb.2013.00410 PMC3872333 24400002

